# Parameterizing Spatial Models of Infectious Disease Transmission that Incorporate Infection Time Uncertainty Using Sampling-Based Likelihood Approximations

**DOI:** 10.1371/journal.pone.0146253

**Published:** 2016-01-05

**Authors:** Rajat Malik, Rob Deardon, Grace P. S. Kwong

**Affiliations:** 1 Department of Mathematics & Statistics, University of Guelph, Guelph, Ontario, Canada; 2 Faculty of Veterinary Medicine, University of Calgary, Calgary, Alberta, Canada; 3 Department of Mathematics & Statistics, University of Calgary, Calgary, Alberta, Canada; Shanxi University, CHINA

## Abstract

A class of discrete-time models of infectious disease spread, referred to as individual-level models (ILMs), are typically fitted in a Bayesian Markov chain Monte Carlo (MCMC) framework. These models quantify probabilistic outcomes regarding the risk of infection of susceptible individuals due to various susceptibility and transmissibility factors, including their spatial distance from infectious individuals. The infectious pressure from infected individuals exerted on susceptible individuals is intrinsic to these ILMs. Unfortunately, quantifying this infectious pressure for data sets containing many individuals can be computationally burdensome, leading to a time-consuming likelihood calculation and, thus, computationally prohibitive MCMC-based analysis. This problem worsens when using data augmentation to allow for uncertainty in infection times. In this paper, we develop sampling methods that can be used to calculate a fast, approximate likelihood when fitting such disease models. A simple random sampling approach is initially considered followed by various spatially-stratified schemes. We test and compare the performance of our methods with both simulated data and data from the 2001 foot-and-mouth disease (FMD) epidemic in the U.K. Our results indicate that substantial computation savings can be obtained—albeit, of course, with some information loss—suggesting that such techniques may be of use in the analysis of very large epidemic data sets.

## Introduction

Modeling the spread of infectious diseases is a research area of great importance to public health and agriculture. Particularly, studies involving data-driven spatial models have recently been used in a number of applications. Work by [1], for example, illustrate the importance of incorporating spatial and temporal data in the mathematical modeling of infectious diseases. Studies have also investigated factors that influence disease persistence/extinction such as infection rate (e.g., [2]). In addition, disease control policies and vaccination policies can be better developed as a result of the understanding of the spread of infectious disease gained from using such models [3]. The increase in computational power over the last several years and the availability of spatio-temporal data have been key factors driving growth in this area of statistics [4].

Significant computational prowess is required for models utilizing large-scale spatial data, such as recent studies performed on the 2001 foot-and-mouth disease (FMD) epidemic in the U.K. (e.g., [5–8]). These can all be considered examples of modeling infectious disease dynamics at the individual-level, although the individuals of concern may vary between studies (e.g., plants, humans, farms, etc.). [8] describe a framework of discrete-time individual-level models (ILMs) that are capable of modeling the spread of infectious diseases in such disease systems. These models can incorporate various heterogeneities within a population; for example, [9] consider the spread of human influenza allowing for vaccination status, age, and the disease status of fellow household occupants in the model. Although ILMs are flexible and intuitive, inference for these and other similar models, especially when dealing with large data sets, can be computationally prohibitive. In fact, even for moderately sized populations, obtaining meaningful results can require running these models for a considerable amount of time.

For such models, parameter estimation is typically carried out in a Bayesian framework via Markov chain Monte Carlo (MCMC) methods (e.g., [10]), wherein the likelihood function (a primary source of the computational problem) is calculated numerous times. An obvious way to reduce the extent of this problem would be to make a simplifying assumption, such as homogeneous mixing (e.g., [11]). However, by allowing for heterogeneity within the population, we hope to draw more sound inferences.

Numerous studies have focused on speeding up the likelihood calculation to reduce the time required to carry out parameter estimation in such models. For example, [8] introduce an approach using a Taylor series expansion of the non-linear spatial infection kernel, allowing for the decomposition of a substantial part of the likelihood function into a small parameter-dependent part and a larger data-dependent part. [12] expand on this by exploring the use of a piecewise linear kernel to carry out the linearization. In both cases, time-saving ensues from the fact that the data-intensive component of the likelihood does not require re-calculation at each step of the MCMC algorithm. However, the resulting model is an approximation of the true model we might actually want to fit. These approaches are also limited to situations where infection event histories of individuals are assumed known.

[13] explore several variations of a random-walk Metropolis algorithm to achieve computational efficiency in the context of infectious disease modeling. Their approach involves pre-calculating and storing quantities that are used repeatedly (something vital to the approaches of [8] and [12]), performing calculations in parallel, and refining the calculation of the likelihood ratio in the Metropolis-Hastings MCMC algorithm.

Other approaches to decreasing computation time in the context of fitting infectious disease models to data—in these cases, homogeneous-mixing models—are based around so-called approximate Bayesian computations, as explored in [14] and [15], and pseudo-marginal approaches, as discussed by [16]. In such approaches, explicit likelihood calculation is completely avoided. An alternative approach is given by [17], not within the context of inference for infectious disease transmission models, but for mixture models. They explore methods basing inference on carefully selected subsamples of data, constructed to provide the most relevant information to the parameters of interest.

In this paper, we consider an approach similar in nature to that of [17] that replaces the likelihood calculation with a faster likelihood approximation based upon data sampling. We also include infection time uncertainty in our modeling via a data augmented Bayesian analysis. The method works by selecting samples from the infected set of individuals at every discrete time point when calculating the infection rate for susceptible individuals, thereby avoiding the need to use the entire infectious set. We show how the resulting approximated likelihood function-based analysis can require significantly less time to carry out and compare the approximate posterior inference to the full Bayesian analysis. Whereas the aforementioned approximate methods of [8] and [12] for parameterizing ILMs cannot be used in a data augmented framework in which infection times are considered unknown, we show how our approach, with careful algorithm development, can allow such uncertainty in the analysis. This is of obvious importance in infectious disease systems because infection event times are very rarely observed with any certainty in practice [18]. We begin by considering a simple random sampling (SRS) approach, followed by a series of spatially-stratified sampling schemes. As a proof of concept, we test our methodology through the use of both simulated data and a relatively small subset of the 2001 UK FMD epidemic data. Note that we consider infectious disease models in a susceptible-infectious-removed (SIR) framework, although extension of the methods to other frameworks (e.g., SEIR) would be relatively straight forward.

Our paper is structured as follows. The *Methodology* section summarizes the general ILM framework of [8], the specific models used in this paper, and the MCMC algorithm used to carry out a full Bayesian MCMC analysis. Our algorithms are presented in the *Sampling-Based Likelihood Approximations* section. The *Epidemic Data* section describes the data used to test our methods and the *Results* section presents our findings. The *Discussion* section concludes this paper and presents possible avenues of future work.

## Methodology

### General Model

We utilize the modeling framework of [8], which defines a class of flexible discrete-time disease transmission models that include covariate information at the individual level. With a finite population of a total of *n* individuals (each individual represented as *i* = 1, …, *n*), we observe epidemic data at discrete time points, *t* = 1, …, *t*_max_, where *t*_max_ is the last observation time. Under a susceptible-infectious-removed (SIR) framework, each individual *i* is in only one of these three states at any given time *t*. If $i\in S_{t}$, then *i* is susceptible to the disease and has not yet contracted it at time *t*; if $i\in I_{t}$, then *i* has contracted the disease and can now infect others at time *t*; and if i∈R, then *i* has been removed from the population at time *t*; e.g., due to recovery combined with acquiring immunity. Once an individual is in this final state, they cannot become infected again or transmit the disease to others. Over the course of the epidemic, individuals move through the three states in the order S→I→R.

As described by [8], the general ILM calculates the probability a susceptible individual *i* will become infectious to the disease at time *t*, and this is given by

$$\begin{array}{c} P_{it}\left(\theta \right)=1-\exp\left[\left\{-\Omega_{S}\left(i\right)\sum_{j\in I_{t}}\Omega_{T}\left(j\right)\kappa \left(i,j\right)\right\}-\epsilon \left(i,t\right)\right], \end{array}$$ (1)

where *Ω*_*S*_(*i*) is a susceptibility function that includes risk factors for individual *i* contracting the disease; *Ω*_*T*_(*j*) is a transmissibility function describing risk factors for individual *j* transmitting the disease to others; *κ*(*i*, *j*) is an infection kernel describing shared risk factors between susceptible and infectious individuals; *ϵ*(*i*, *t*) describes external infectious pressure not explained by the rest of the model and is commonly referred to as the ‘sparks term’; and ***θ*** is the set of ILM parameters we want to estimate.

### Spatial ILM

In this section, we present a simplified version of the general ILM such that *Ω*_*S*_(*i*) = *α*, *Ω*_*T*_(*j*) = 1, and $\kappa \left(i,j\right)=d_{ij}^{-\beta }$. Here, *d*_*ij*_ represents the Euclidean distance between susceptible *i* and infectious *j* and *β* represents the power law rate of decay. We also set the sparks term, *ϵ*(*i*, *t*) = 0. We refer to this model as the Spatial ILM. Under this model, the probability of infection for susceptible *i* at time *t* is given by

$$\begin{array}{c} P_{it}\left(\theta \right)=1-\exp\left[-\alpha \sum_{j\in I_{t}}d_{ij}^{-\beta }\right],\;\alpha >0,\beta >0. \end{array}$$ (2)

### FMD-ILM

We also modify the general ILM in order to model data from the 2001 U.K. FMD epidemic. Using a simplified version of the model found in [8] and by modeling at the farm-level, we can determine the probability that susceptible farm *i* is infected at time *t* using

$$\begin{array}{c} P_{it}\left(\theta \right)=1-\exp\left[\left(-\left(\alpha_{s}N_{i}^{s}+\alpha_{c}N_{i}^{c}\right)\sum_{j\in I_{t}}\left(\phi_{s}N_{j}^{s}+\phi_{c}N_{j}^{c}\right)d_{ij}^{-\beta }\right)-\epsilon \right],\\ \alpha_{c}>0,\phi_{s}>0,\phi_{c}>0,\beta >0,\epsilon >0, \end{array}$$ (3)

where *α*_*s*_ and *α*_*c*_ are susceptibility parameters and *ϕ*_*s*_ and *ϕ*_*c*_ are transmissibility parameters, for sheep and cattle, respectively. The terms $N_{x}^{s}$ and $N_{x}^{c}$ represent the number of sheep and cattle on farm *x*, respectively. To avoid identifiability issues, we set *α*_*s*_ = 1 × 10^−7^, which is an arbitrary constant reference level and is not estimated. Once again, the power-law kernel, $\kappa \left(i,j\right)=d_{ij}^{-\beta }$, is used with *d*_*ij*_ being the Euclidean distance between farms. The sparks terms is set as a constant such that *ϵ*(*i*, *t*) = *ϵ*, which represents a constant infectious pressure from outside the study area. We refer to Model 3 as our FMD-ILM.

### Bayesian Computation

Our parameter estimation is here carried out under a Bayesian framework. Assuming known infection and removal times, the likelihood function for ILMs is the product of all infection and non-infection events over the entire observed epidemic period (*t* = 1, …, *t*_max_), and is given by

$$\begin{array}{c} \pi \left(x|\theta \right)=\prod_{t=1}^{t_{\max}}\left[\prod_{i\in S_{t+1}}\left(1-P_{it}\left(\theta \right)\right)\prod_{i\in I_{t+1}\backslash I_{t}}P_{it}\left(\theta \right)\right], \end{array}$$ (4)

where **x** is the observed epidemic data set (including the infection times), $S_{t+1}$ is the set of all susceptible individuals at time *t* + 1, and $I_{t+1}\backslash I_{t}$ is the set of newly infectious individuals at time *t* + 1. Using our likelihood function and by placing a prior, *π*(***θ***), on our parameter set, ***θ***, we can obtain the posterior distribution, *π*(***θ***|**x**), up to a constant of proportionality. To explore the posterior distribution, we can use the random-walk Metropolis Hastings (RWMH) algorithm [19–21].

**Fig 1:**
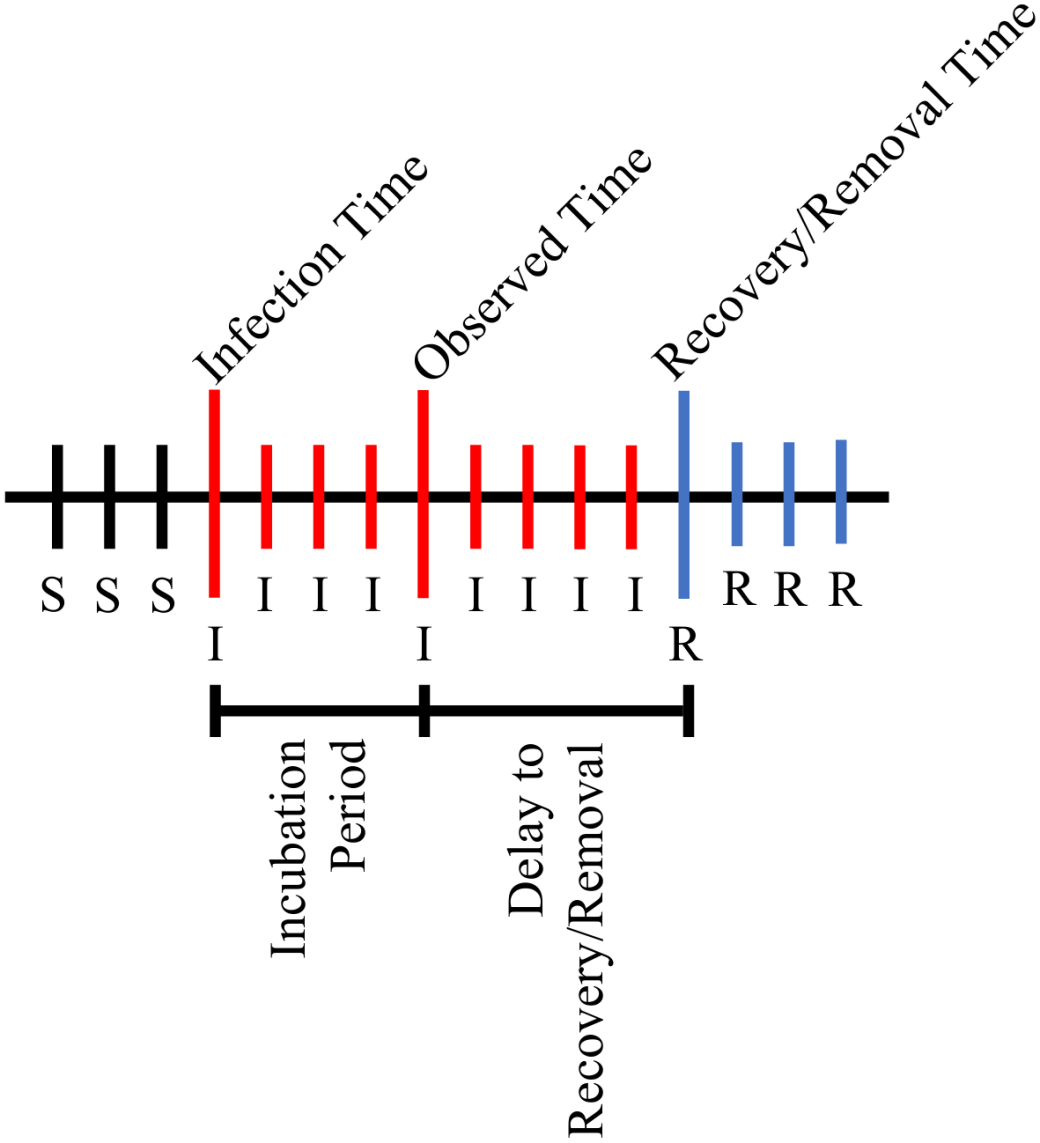
Average infectious period under the simulation study. Illustration of the average infectious period under the simulation study. The average incubation period is 3 days, and the average delay to disease recovery and removal from the population is 4 days. The ‘S’ symbol indicates the individual is susceptible to the disease at that time point and the ‘R’ symbol indicates the individual has recovered from the disease and has been removed from the population at that time point.

We assume here a disease system such as foot-and-mouth as seen in the U.K. in 2001, in which the disease is reported after infection and then individuals are later removed from the population through some intervention (see Fig 1). Thus, we assume that removal times are known and fixed (although this assumption could quite easily be relaxed—see Discussion). However, we do not assume to know when individuals become infectious and so utilize Bayesian data augmentation, treating the infection times as unknown nuisance parameters (see also sections *MCMC Algorithm* and *Data Augmentation*). We determine the infection time indirectly by inferring the incubation period (the time between infection and disease reporting/diagnosis/observation) of each individual. Given the aforementioned assumption that removal times are known, the incubation period thus defines the infection time for each individual. The incubation period, plus a further delay until removal (e.g., through quarantine or animal culling), thus define the infectious period.

We denote the unknown incubation periods $Z=(Z_{1},Z_{2},\ldots ,Z_{v})$, where *v* is the number of infected individuals in [1, *t*_max_], and assume $Z_{c}\overset{\text{i.i.d.}}{∼}\text{DExp}\left(\lambda_{z}\right)$ (discretized exponential distribution), where the rate parameter, *λ*_*z*_, is also to be estimated. We augment the model parameter set to include Z. Under the spatial ILM, our parameter vector is thus $\theta^{+}=\{\alpha ,\beta ,\lambda_{z},Z\}$; and under the FMD-ILM, the parameter set is $\theta^{+}=\{\alpha_{c},\phi_{s},\phi_{c},\beta ,\epsilon ,\lambda_{z},Z\}$.

In general, we define an augmented parameter set, $\theta^{+}=\left(\theta ,Z\right)$, and assuming independence between ***θ*** and Z derive the posterior distribution up to proportionality as

$$\begin{array}{ccc} \pi (\theta^{+}|x^{-}) & \propto & \pi \left(x^{-}|\theta^{+}\right)\pi \left(\theta^{+}\right)\\ & = & \pi \left(x^{-}|\theta ,Z\right)\pi \left(\theta \right)\pi \left(Z\right)\\ & = & \pi \left(x^{-},Z|\theta \right)\pi \left(\theta \right), \end{array}$$ (5)

where **x**^−^ is the epidemic data not including infection times and *π*(***θ***^+^|**x**^−^) is sampled from using Metropolis-Hastings MCMC. Here, we use an independence sampler to update *λ*_*z*_ and Z and random-walk updates for other model parameters. Note that we are only indirectly estimating the infectious period distribution and not using any prior information on the infectious period. This framework could easily be changed, of course, to fit the requirements of other disease systems.

### MCMC Algorithm

Here, we outline our MCMC algorithm to update the data augmented parameter set, ***θ***^+^, and obtain realizations from the posterior distribution, *π*(***θ***^+^|**x**^−^), in order to carry out a full gold-standard Bayesian analysis. For our MCMC procedure, we break down our augmented parameter set and let Θ contain the same set of parameters as ***θ*** but without rate parameter *λ*_*z*_; i.e., Θ = {*θ*_1_, …, *θ*_*b*_}, where *b* = |Θ| (the number of parameters in Θ). Hence, for our MCMC algorithm below, we specify our augmented parameter set as $\Phi =(\Theta ,\lambda_{z},Z)$. There are a total of v=|Z| parameters in Z, and a total of *d* = *b* + *v* + 1 parameters in Φ. We define *θ*_*w*_ as the *w*^th^ parameter in Θ and $Z_{c}$ as the *c*^th^ parameter in Z. Let *r* be a counter for the number of MCMC iterations and let $\lambda_{z}^{r}$ represent the *r*^th^ iteration of *λ*_*z*_. The MCMC algorithm is as follows:

1. Let *r* = *r* + 1.
2. Update Z:
  - Let *c* = 1.
  - Given the current position, $Z_{c}^{r}$, generate a new value, $Z_{c}^{r+1}∼\text{DExp}\left(\lambda_{z}^{r}\right)$, using the inverse transform method.
  - Calculate the acceptance probability,
    $$\begin{array}{c} A=\min\left(1,\frac{\pi (\Theta^{r},\lambda_{z}^{r},Z_{1}^{r+1},Z_{2}^{r+1},\cdots ,Z_{c}^{r+1},Z_{c+1}^{r},Z_{c+2}^{r},\cdots ,Z_{v}^{r}|x)}{\pi (\Theta^{r},\lambda_{z}^{r},Z_{1}^{r+1},Z_{2}^{r+1},\cdots ,Z_{c}^{r},Z_{c+1}^{r},Z_{c+2}^{r},\cdots ,Z_{v}^{r}|x)}\right). \end{array}$$
  - With probability A, accept $Z_{c}^{r+1}$. Otherwise, set $Z_{c}^{r+1}=Z_{c}^{r}$.
  - Let *c* = *c* + 1.
  - If *c* ≤ *v*, then repeat from step 2b.
  - If *c* > *v*, then continue to step 3.
3. Update Θ:
  - Let *w* = 1.
  - Given the current position, $\theta_{w}^{r}$, generate a new value such that $\theta_{w}^{r+1}=\theta_{w}^{r}+s$, where *s* ∼ *U*[−*g*_*w*_, *g*_*w*_], $g_{w}\in R^{+}$, and $\theta_{w}^{r+1}>0$.
  - Calculate the acceptance probability,
    $$\begin{array}{c} A=\min\left(1,\frac{\pi (\theta_{1}^{r+1},\theta_{2}^{r+1},\cdots ,\theta_{w}^{r+1},\theta_{w+1}^{r},\theta_{w+2}^{r},\cdots ,\theta_{b}^{r},\lambda_{z}^{r},Z^{r+1}|x)}{\pi (\theta_{1}^{r+1},\theta_{2}^{r+1},\cdots ,\theta_{w}^{r},\theta_{w+1}^{r},\theta_{w+2}^{r},\cdots ,\theta_{b}^{r},\lambda_{z}^{r},Z^{r+1}|x)}\right). \end{array}$$
  - With probability A, accept $\theta_{w}^{r+1}$. Otherwise, set $\theta_{w}^{r+1}=\theta_{w}^{r}$.
  - Let *w* = *w*+1.
  - If *w* ≤ *b*, then repeat from step 3b.
  - If *w* > *b*, then continue to step 4.
4. Update *λ*_*z*_ via the independence sampler:
  - Given the current position, $\lambda_{z}^{r}$, generate a new value such that $\lambda_{z}^{r+1}∼\Gamma \left(f_{1},f_{2}\right)$, where *f*_1_ is a shape parameter and *f*_2_ is a rate parameter.
  - Calculate the acceptance probability,
    $$\begin{array}{c} A=\min\left(1,\frac{\pi (\Theta^{r+1},\lambda_{z}^{r+1},Z^{r+1}|x)}{\pi (\Theta^{r+1},\lambda_{z}^{r},Z^{r+1}|x)}\right). \end{array}$$
  - With probability A, accept $\lambda_{z}^{r+1}$. Otherwise, set $\lambda_{z}^{r+1}=\lambda_{z}^{r}$.
5. Repeat from step 1 until a sufficiently large sample of realizations has been obtained.

## Sampling-Based Likelihood Approximations

As stated previously, the full likelihood calculation for ILMs can be computationally taxing. Our focus here is on the key problem of calculating the infectious pressure,

$$\begin{array}{c} X_{it}=\sum_{j\in I_{t}}\Omega_{T}\left(j\right)\kappa \left(i,j\right), \end{array}$$

for each individual $i\in S_{t+1}$ and $i\in I_{t+1}\backslash I_{t}$ at each time point for which data are observed. The problem worsens when we attempt to incorporate infection time (or incubation period) parameters via data augmentation. In doing so, we increase the number of parameters and, thus, the number of parameter updates in each MCMC iteration.

We propose to alleviate this problem by estimating $X_{it}$ by sampling from the infectious set $I_{t}$ at each time point that data are observed. We begin this section by outlining the need to organize all infectious individuals into a matrix that can be updated in an efficient manner as the incubation period (and, thus, infection time) parameters are updated as part of the data-augmented MCMC. We then detail our sampling algorithms in such a data-augmented context. The two algorithms considered here allow for an SRS approach and a spatially-stratified sampling scheme, respectively, for sampling from the $I_{t}$ sets.

### Simple Random Sampling Algorithm

For the SRS method, calculating each *P*_*it*_(***θ***) (or 1 − *P*_*it*_(***θ***)) in the likelihood is achieved by replacing the full set of infectious individuals $I_{t}$ with a set ${\hat{I}}_{t}$ obtained through SRS with replacement from the set $I_{t}$, and scaling by the empirical sampling proportion, ${\hat{\rho }}_{t}$. This method is shown to severely reduce the computational time required to calculate *P*_*it*_ in the likelihood function. When $I_{t}$ is ‘small’ (i.e., $|I_{t}\left|\leq q\right|$), we do not sample and use the entire infectious set. Here, we set *q* = 10 because the time savings would be negligible for *q* ≤ 10 simply due to the overhead associated with sampling. The infectious pressure is approximated as

Xit≃X^it=ρ^t-1∑j∈ItΩT(j)κ(i,j)|It|≤qρ^t-1∑j∈I^tΩT(j)κ(i,j)|It|>q

and thus the approximation of our original probability of infection is

$$\begin{array}{c} P_{it}\left(\theta \right)≃{\hat{P}}_{it}\left(\theta \right)=1-\exp\left[\left\{-\Omega_{S}\left(i\right){\hat{X}}_{it}\right\}-\epsilon \left(i,t\right)\right], \end{array}$$ (6)

which we refer to as the SRS-ILM. Initially, we will assume that all infection times (as well as removal times) are known.

We now define notation relevant to our infection matrix, M, of dimension *n* × *t*_max_. The elements of M take the form of integer identification numbers for each farm and thus, each column, $M[\cdot ,t_{1},\ldots ,t_{\max}]$ consists of an arbitrary ordering of farm IDs indicating their infection times, followed by a series of zeros in the remaining elements of the column. We also store the length of each column of the matrix up until the presence of empty cells, defined as $\ell_{t}=\left|M\left[\cdot ,t\right]\right|$. We use the notation M[B,C] to represent the farm ID located in row *B* and time column *C* in matrix M. We also use the notation DU[a,b] to refer to a discrete uniform on [*a*, *b*], a,b∈Z, i.e., a distribution consisting of equally sized point masses on all integers within the interval [*a*, *b*]. To calculate the likelihood function, we then use the following algorithm:

1. Let ${\hat{L}}_{t}=0\;\forall \;t=1,\ldots ,t_{\max}$ and set *t* = 1.
2. If ℓ_*t*_ ≤ *q*, calculate the full likelihood component for time *t*,
  $$\begin{array}{c} {\hat{L}}_{t}=\prod_{i\in S_{t+1}}\left(1-P_{it}\left(\theta \right)\right)\prod_{i\in I_{t+1}\backslash I_{t}}P_{it}\left(\theta \right), \end{array}$$
  and go to step 6.
  Else, if ℓ_*t*_ > *q*, let *ξ* = *ρ*_*t*_ℓ_*t*_ and continue to step 2.
3. Let *c* = 0 and ${\hat{v}}_{t}$ be a set of “empty” vectors of length to be determined by the algorithm.
4. Let *c* = *c* + 1.
5. If *c* ≤ *ξ*, then simulate $U∼\text{DU}[1,\ell_{t}]$, let ${\hat{v}}_{t}\left[c\right]=M\left[t,U\right]$, and return to step 3.
  If *c* > *ξ*, then let ${\hat{I}}_{t}$ be the set containing all *c* − 1 elements of ${\hat{v}}_{t}$ and continue to step 5.
6. Calculate the approximated likelihood component for time *t*,
  L^t=∏i∈St+1exp-ΩS(i)X^it-ϵ(i,t)×=∏i∈I^t+1\I^t1-exp-ΩS(i)X^it-ϵ(i,t),
  where ${\hat{X}}_{it}={\hat{\rho }}_{t}^{-1}\sum_{j\in {\hat{I}}_{t}}\Omega_{T}\left(j\right)\kappa \left(i,j\right)$, as before, and ${\hat{\rho }}_{t}=\frac{c-1}{\ell_{t}}$.
7. Let *t* = *t*+1.
  If *t* < *t*_max_, then go to step 1.
  Else, if *t* = *t*_max_, then calculate the approximated likelihood function,
  $$\begin{array}{c} \hat{\pi }\left(x|\theta \right)=\prod_{t=1}^{t_{\max}}{\hat{L}}_{t}. \end{array}$$

### Spatially-Stratified Sampling Algorithm

In this section, we consider grouping individuals into strata based on their *x*−*y* coordinates. From here, we sample only a proportion of the infectious set from each stratum at each time point *t* when calculating ${\hat{P}}_{it}\left(\theta \right)$. Let *k* represent the index for each stratum up to a total of *m* strata and let

Z^itk≃ρ^tk-1∑j∈ItkΩT(j)κ(i,j)|Itk|≤qρ^tk-1∑j∈I^tkΩT(j)κ(i,j)|Itk|>q

be the estimate of the infectious pressure exerted on susceptible individual *i* from stratum *k* at time *t*. Here, ${\hat{\rho }}_{tk}$ is the empirical sampling proportion of the sampled infectious set for the stratum, $I_{tk}$ is the complete set of infectious individuals in strata *k* at time *t*, and ${\hat{I}}_{tk}$ is the randomly sampled set of infectious individuals obtained via SRS (with replacement) from strata *k* with empirical sampling proportion ${\hat{\rho }}_{tk}$. The sum of infectious pressures from each stratum exerted on individual *i* at time *t* is referred to as the total infectious pressure and calculated as

$$\begin{array}{c} {\hat{Z}}_{it}=\sum_{k=1}^{m}{\hat{Z}}_{itk}. \end{array}$$

As before, for small infectious sets, i.e., $|I_{tk}|\leq q$, we use the entire infectious set and do not sample. Under a spatial-stratification scheme, we use *q* = 5. Thus, the approximation of the probability of infection is

$$\begin{array}{c} P_{it}\left(\theta \right)≃{\hat{P}}_{it}\left(\theta \right)=1-\exp\left[\left\{-\Omega_{S}\left(i\right){\hat{Z}}_{it}\right\}-\epsilon \left(i,t\right)\right], \end{array}$$ (7)

which is substituted into our likelihood function. We refer to this model as the SSS-ILM.

We consider a three-dimensional infection matrix, Q, with dimensions *t*_max_ × *m* × *n* that contain elements corresponding to integer identification numbers for each farm. We use the notation Q[B,C,D] to refer to the farm ID located at time *B*, stratum *C*, and cell *D* within matrix Q. We also define a two-dimensional matrix, W, with dimensions *t*_max_ × *m* that contain the number of infectious individuals in each stratum at every time point (up until the presence of empty cells). Thus, for each combination of *t* = 1, …, *t*_max_ and *k* = 1, …, *m*, W[t,k]=|Q[t,k,·]|, where W[t,k] represents the number of infectious individuals at time *t*, in stratum *k*. We use the following algorithm to calculate the approximated likelihood function under the spatial stratification scheme:

1. Let ${\hat{L}}_{tk}=0\;\forall \;t=1,\ldots ,t_{\max}$ and *k* = 1, …, *m*.
  Set *t* = 1, *k* = 1 and $\ell_{tk}=W\left[t,k\right]$.
2. If ℓ_*tk*_ ≤ *q*, calculate the likelihood component for strata *k* at time *t*,
  $$\begin{array}{c} {\hat{L}}_{tk}=\prod_{i\in S_{(t+1),k}}\left(1-P_{it,k}\left(\theta \right)\right)\prod_{i\in I_{(t+1),k}\backslash I_{tk}}P_{it,k}\left(\theta \right), \end{array}$$
  and go to step 7.
  Else, if ℓ_*tk*_ > *q*, let *ξ* = *ρ*_*tk*_ℓ_*tk*_ and continue to step 2.
3. Let *c* = 0 and ${\hat{v}}_{tk}$ be a set of “empty” vectors of length to be determined by the algorithm.
4. Let *c* = *c* + 1.
5. If *c* ≤ *ξ*, then simulate $U∼\text{DU}[1,\ell_{tk}]$, let ${\hat{v}}_{tk}\left[c\right]=v_{tk}\left[U\right]$, and return to step 3.
  If *c* > *ξ*, then let ${\hat{I}}_{tk}$ be the set containing all *c* − 1 elements of ${\hat{v}}_{tk}$ and continue to step 5.
6. Calculate the approximated likelihood component for strata *k* at time *t*,
  L^tk=∏i∈S(t+1),kexp-ΩS(i)Z^itk-ϵ(i,tk)×=∏i∈I^(t+1),k\I^tk1-exp-ΩS(i)Z^itk-ϵ(i,tk),
  where ${\hat{Z}}_{itk}≃{\hat{\rho }}_{tk}^{-1}\sum_{j\in {\hat{I}}_{tk}}\Omega_{T}\left(j\right)\kappa \left(i,j\right)$, as defined earlier, and ${\hat{\rho }}_{tk}=\frac{c-1}{\ell_{tk}}$.
7. Let *k* = *k* + 1.
  If *k* ≤ *m*, then go to step 1.
  Else, if *k* > *m*, continue and calculate the approximated likelihood function for time *t*,
  $$\begin{array}{c} {\hat{L}}_{t}=\prod_{k=1}^{m}{\hat{L}}_{tk}. \end{array}$$
8. Let *t* = *t*+1.
  If *t* ≤ *t*_max_, then reset *k* = 1 and go to step 1.
  Else, if *t* > *t*_max_, then calculate the approximated likelihood function:
  $$\begin{array}{c} \hat{\pi }\left(x|\theta \right)=\prod_{t=1}^{t_{\max}}{\hat{L}}_{t}. \end{array}$$

### Data Augmentation

Because infection times/incubation periods are unknown and can change during the incubation period MCMC update, their infectious period can become longer or shorter; recall that, in our framework, the removal times remain constant. Thus, as each individual’s infection time changes, we continually need to update our infection matrix to reflect the current infection times. For computational reasons, however, it is vital that the infection matrix, Q, be updated in as efficient a manner as possible (and certainly not reconstructed from scratch) as these data-augmented parameters change.

As an example, say individual *i*_3_’s current infectious period is from *t*_2_ → *t*_4_, and we are carrying out simple random sampling (i.e., no stratification). During the update process, the infection time increases by one and now *i*_3_ is only infectious during the period of *t*_3_ → *t*_4_. Below, we illustrates the matrix update process, and use 0s to represent empty cells. Each column represents a time point and the number of individuals infected at each time is displayed underneath the matrix. The first matrix shows the current infection times (before the update) for all individuals, including *i*_3_. The second matrix shows that at time column *t*_2_, *i*_3_ is removed from its current position and replaced with a temporarily empty cell. The final matrix shows that *i*_5_, which is the *last* individual in the *t*_2_ column, is moved to *i*_3_’s old position (now *i*_5_’s new position) and *i*_5_’s old position is replaced with an empty cell. At each state, the number of elements in each column is also updated.

State before update:

|It|(t=1t=2t=3t=4t=5…t=tmax-1t=tmaxi1i1i2i2i2…in−1in0i2i3i3i6…in00i3i4i4i7…000i4i5i6i8…000i5i6i7i9…0000i7i80…00⋮⋮⋮⋮⋮…⋮⋮00000…0015665…21)

Intermediate step: (removing *i*_3_ from the *t*_2_ column)

|It|(t=1t=2t=3t=4t=5…t=tmax-1t=tmaxi1i1i2i2i2…in−1in0i2i3i3i6…in000i4i4i7…000i4i5i6i8…000i5i6i7i9…0000i7i80…00⋮⋮⋮⋮⋮…⋮⋮00000…001*665…21)

Final state of the matrix: (following movement of last element of *t*_2_ column)

|It|(t=1t=2t=3t=4t=5…t=tmax-1t=tmaxi1i1i2i2i2…in−1in0i2i3i3i6…in00i5i4i4i7…000i4i5i6i8…0000i6i7i9…0000i7i80…00⋮⋮⋮⋮⋮…⋮⋮00000…0014665…21)

Then, if the change in infection time results in an individual being infected at a time point at which they were not previously, then they are simply added at the first zero in the particular column. So, for example in this case, if at the next MCMC iteration *i*_3_’s infection time changed such that they returned to having an infectious period from *t*_2_ → *t*_4_, then a new matrix would be formed:

New state of the matrix:

|It|(t=1t=2t=3t=4t=5…t=tmax-1t=tmaxi1i1i2i2i2…in−1in0i2i3i3i6…in00i5i4i4i7…000i4i5i6i8…000i3i6i7i9…0000i7i80…00⋮⋮⋮⋮⋮…⋮⋮00000…0015665…21)

By following these methods, we avoid the problem of ending up with zeros in the middle of columns representing each time point (which would require searching, keeping track of where non-zeros are, or recompiling the matrix), and we only require sampling from the first $|I_{t}|$ of each column of the matrix. In our MCMC update, infection times, and thus periods, increment or decrement only by one time point at each MCMC iteration, and are considered in single parameter updates. However, this scheme can easily be extended to allow for updates of larger magnitude, or indeed block updates.

Under the spatial stratification sampling schemes, the same method of adding to the first non-zero of a column, and switching a zero in the middle of a non-zero section of a column with the last non-zero of said column, is used. However, we are of course now working with columns in the 3-dimensional rather than 2-dimensional Q.

## Epidemic Data

To demonstrate the effectiveness of our sampling methods, we apply them to real and simulated data. Here, we describe these data in some detail, beginning with the simulated epidemic data and followed by the 2001 U.K. FMD epidemic.

### Simulated Data

Using the Spatial ILM, we generated ten epidemics. The chosen population is of size *n* = 625 spread out evenly on a 25 × 25 grid, 1 unit apart in the *x* and *y* planes. Our susceptibility parameter is set to *α* = 1.4 and our power law spatial parameter is set to *β* = 2.3. We generated the incubation period from an exponential distribution with rate parameter $\lambda_{z}=\frac{1}{3}$, giving an average incubation period of 3 days. The period from disease diagnosis to disease recovery and removal from the population was also generated from an exponential distribution with rate parameter $\lambda_{w}=\frac{1}{4}$, resulting in an average delay period of 4 days. Thus, the total infectious period, on average, is 7 days. Fig 1 illustrates the average infectious and non-infectious periods. In our modeling, we assume we know removal times but not the incubation period and thus estimate it via data augmentation. There is also an implicit assumption that the observation time occurs before removal.

### FMD Data

We also implement our sampling-based parameterization methods on data from the 2001 U.K. FMD epidemic. We used a subset of the epidemic data, which was from the county of Cumbria located in North West England and consisted of 1,636 individual farms. According to [22], sheep and cattle farms accounted for almost all cases of the FMD outbreak in 2001 in the U.K. We consider farms to be the “individual”-level at which we are modeling and use cattle and sheep populations within farms as covariates in our model. We treat the disease diagnosis times recorded by veterinarians and epidemiologists who were on the ground during the outbreak as observed infection times. The times when animals were culled were also recorded and we treat these as the removal times in our modeling framework. We estimate the incubation periods (and the infection times indirectly) through Bayesian data augmentation. For some farms, disease diagnosis times were not recorded and so we assume these farms transition from state S→R on their cull date. In total, 730 infections were recorded in our data set. Refer to, for example, [8], for a more detailed description of the U.K. 2001 data set.

Note that most models for FMD assume an SEIR framework, with a latent, non-infectious state before infectiousness. We simplify our model for the purposes of illustration of our method, and—as mentioned in the discussion—extension to an SEIR framework would be relatively straightforward.

### Priors

For both data sets, all ILM parameters, except for *λ*_*z*_, are assigned independent, vague marginal priors under the assumption of weak prior knowledge. The marginal priors chosen here are positive, half-normal distributions with mode 0 and a ‘large’ variance of 10^5^.

The marginal prior choice for *λ*_*z*_, the incubation period rate parameter, is a Gamma distribution such that *λ*_*z*_ ∼ Γ(3, 9). This prior suggests an average incubation period of 3 days, which is the same as the incubation period from the simulated data. For the FMD epidemic, this prior may, of course, be misspecified because we do not know the actual incubation period.

## Results

In this section, we present the results of our analyses. All computations were performed on an Apple Mac Pro with two 6-core Intel Xeon 2.93 GHz processors with 12 GB of RAM.

### Simulation Study

**Fig 2:**
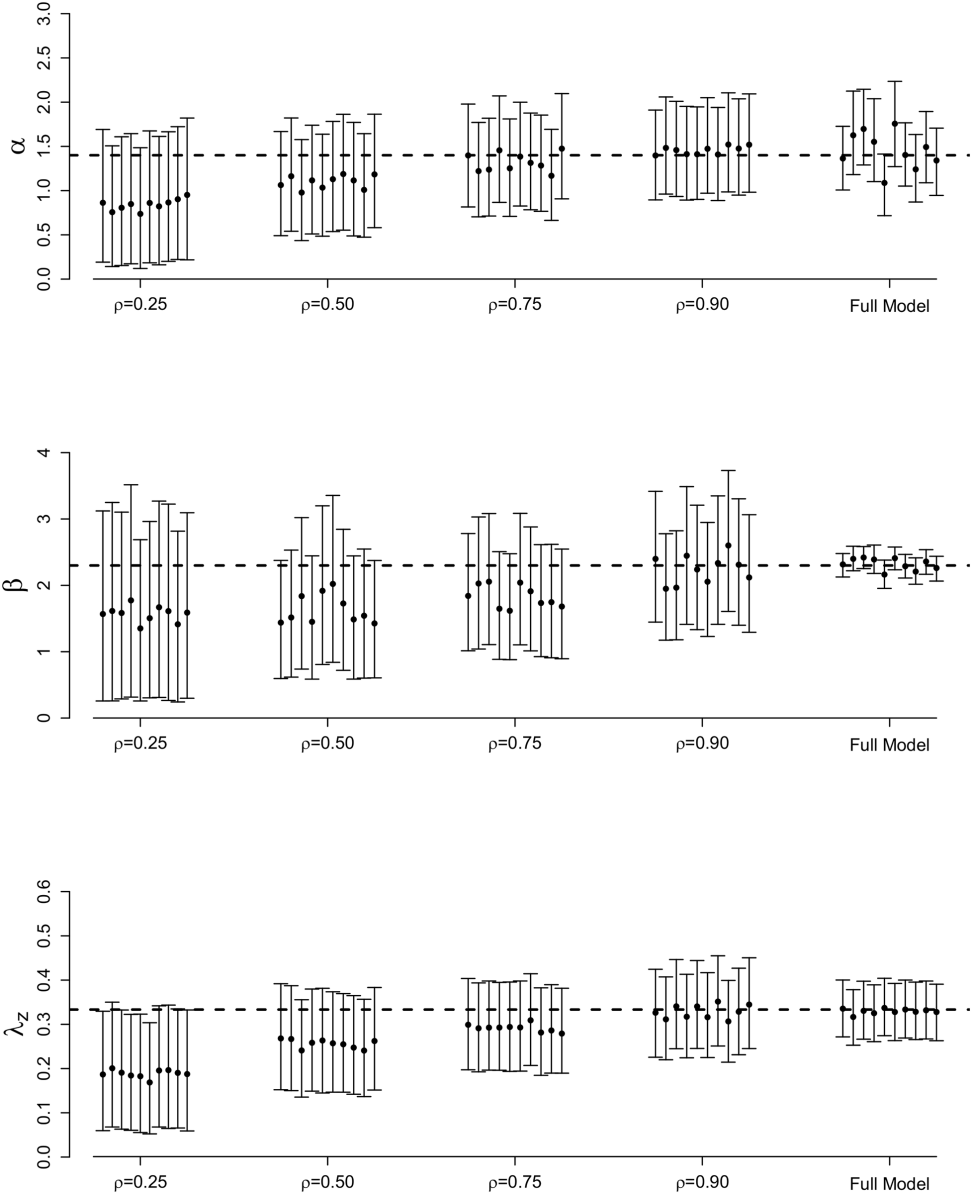
Posterior results for full MCMC and SRS methods. Posterior means and 95% credible intervals for *α*, *β*, and *λ*_*z*_ for the full MCMC and SRS methods for 10 different epidemics simulated from the data augmented spatial ILM with varying sampling proportions. The dashed, horizontal lines represent the true parameter values: *α* = 1.4, *β* = 2.3, and $\lambda_{z}=\frac{1}{3}$, with a population of size *n* = 625.

**Fig 3:**
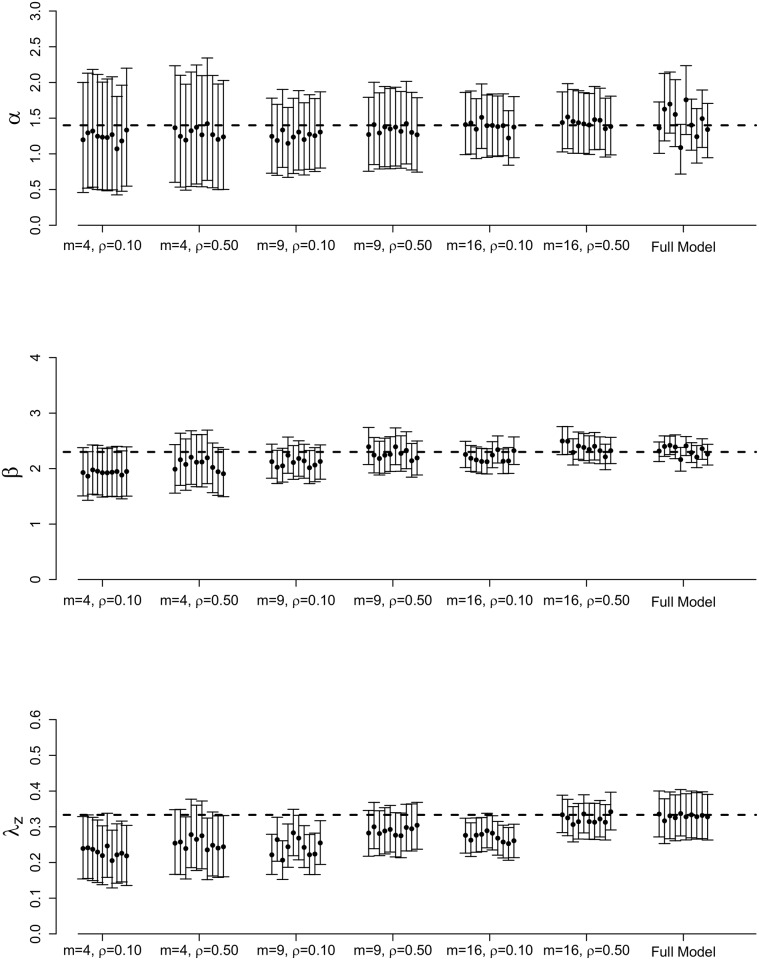
Full posterior results for full MCMC and spatial stratification methods. Posterior means and 95% credible intervals for *α*, *β*, and *λ*_*z*_ for the full MCMC and spatial stratification methods for 10 different epidemics simulated from the data augmented spatial ILM with varying values for *m* and *ρ*. The dashed, horizontal lines represent the true parameter values: *α* = 1.4, *β* = 2.3, and $\lambda_{z}=\frac{1}{3}$, with a population of size *n* = 625.

Figs 2 and 3 illustrate the posterior means and 95% credible intervals for each ILM parameter, for each of the 10 epidemics simulated. Averages of these results over the ten epidemic data sets are shown in S1 Table.

As expected, bias for all parameters decreases as the sampling proportion, *ρ*, increases under the SRS technique. Posterior variance also decreases as *ρ* increases, leading to tighter credible intervals. It also appears to be more difficult to estimate the spatial parameter *β* using an SRS scheme with precision approaching that seen under the full MCMC analysis than is the case with *α* and *λ*_*z*_.

Introducing spatial stratification in our sampling scheme appears to lead to increased posterior accuracy (Fig 3). Under these results (shown for *ρ* = 0.10 and *ρ* = 0.50), as the number of strata, *m*, increases, posterior variance and bias decrease and, thus, credible intervals are tighter. The posterior means also tend to be closer to the true parameter values. We also observe that spatial stratification is less sensitive to different *ρ* values tested and more sensitive to different values for *m*. For example, in Fig 3, the posterior results for *α* under *m* = 4 show almost no change for *ρ* = 0.10 versus *ρ* = 0.50. However, increasing the number of strata to *m* = 9 does increase posterior accuracy compared to *m* = 4. Under both values of *ρ*, we are able to obtain more posterior accuracy under the spatial stratification scheme than the simple random sampling scheme, demonstrating the advantage that including spatial stratification presents.

However, although spatial stratification appears more desirable in terms of posterior approximation, we must also consider the computation time required for the MCMC to run. Table 1 displays the average computation time (in hours) of each of our sampling methods. We observe that it takes approximately 92.64 hours to run 20,000 MCMC iterations of the true model without any data sampling. However, if we introduce SRS and set *ρ* = 0.25, we notice a drastic reduction in computation time; the MCMC takes only 36.48 hours to run. The computation time increases when *ρ* increases, as expected. The same is true if we consider spatial stratification in our sampling scheme. We see that smaller values of *m* yield faster run times, but the posterior approximation improves with larger *m*. Obviously, in practice, a trade off between posterior accuracy and computation time would be required.

**Table 1:** Average computation time for the simulation studies.

*ρ*	m	Computation Time (hours)
—	—	92.64
0.25	—	36.48
0.50	—	47.76
0.75	—	59.28
0.90	—	75.12
0.10	4	46.88
0.50	4	56.16
0.10	9	66.24
0.50	9	71.52
0.10	16	82.32
0.50	16	88.56

Average computation run times (in hours) of fitting the data augmented spatial ILM, SRS-ILM, and the SSS-ILM to 10 different simulated epidemics. These epidemics were simulated using ILM parameters *α* = 1.4, *β* = 2.3, $\lambda_{z}=\frac{1}{3}$, and *n* = 625.

### FMD Model Fitting Results

**Fig 4:**
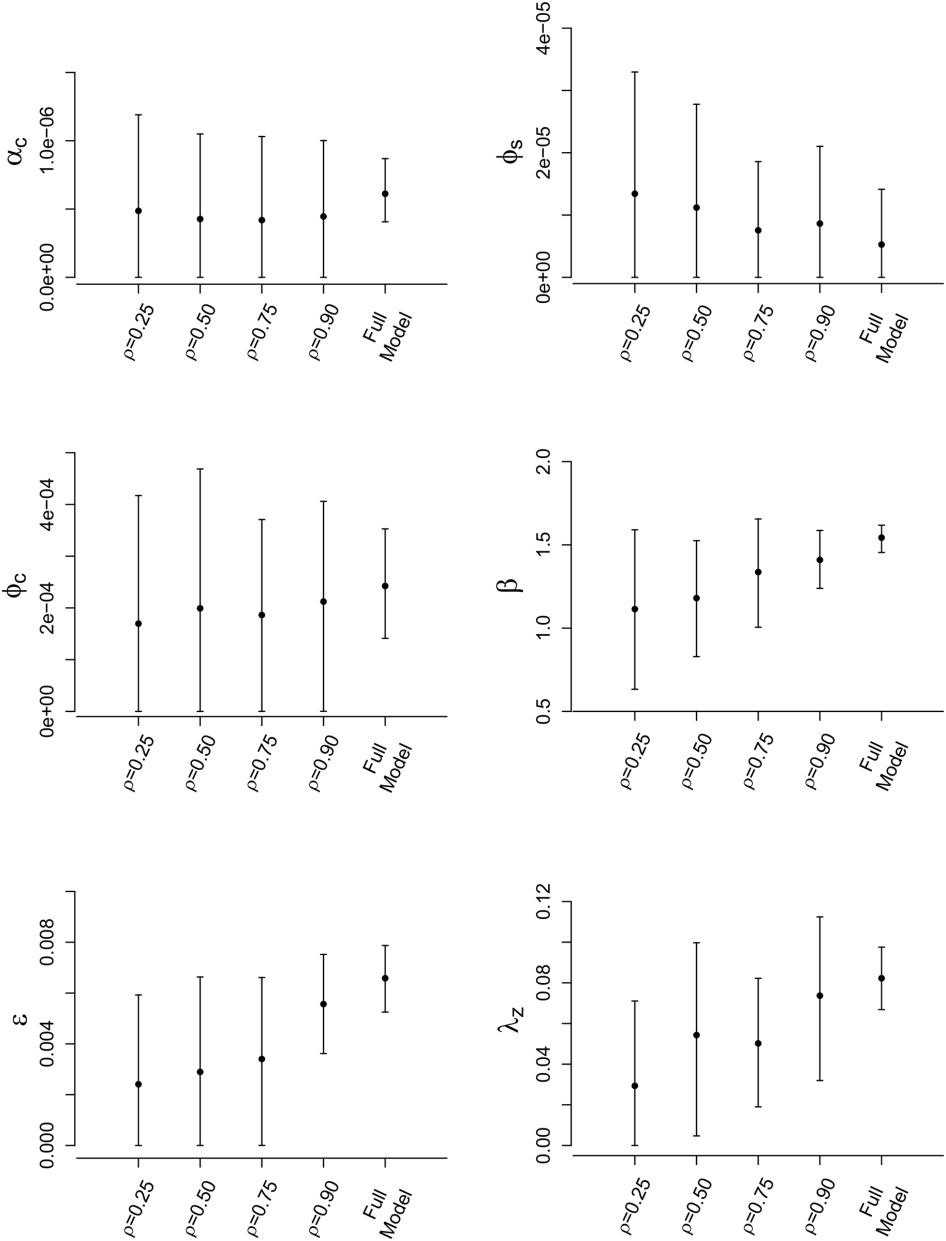
Posterior results for FMD-ILM using the SRS method. Posterior means and 95% credible intervals for all parameters of the data augmented FMD-ILM under the SRS method. The results are compared to the full model to assess accuracy.

**Fig 5:**
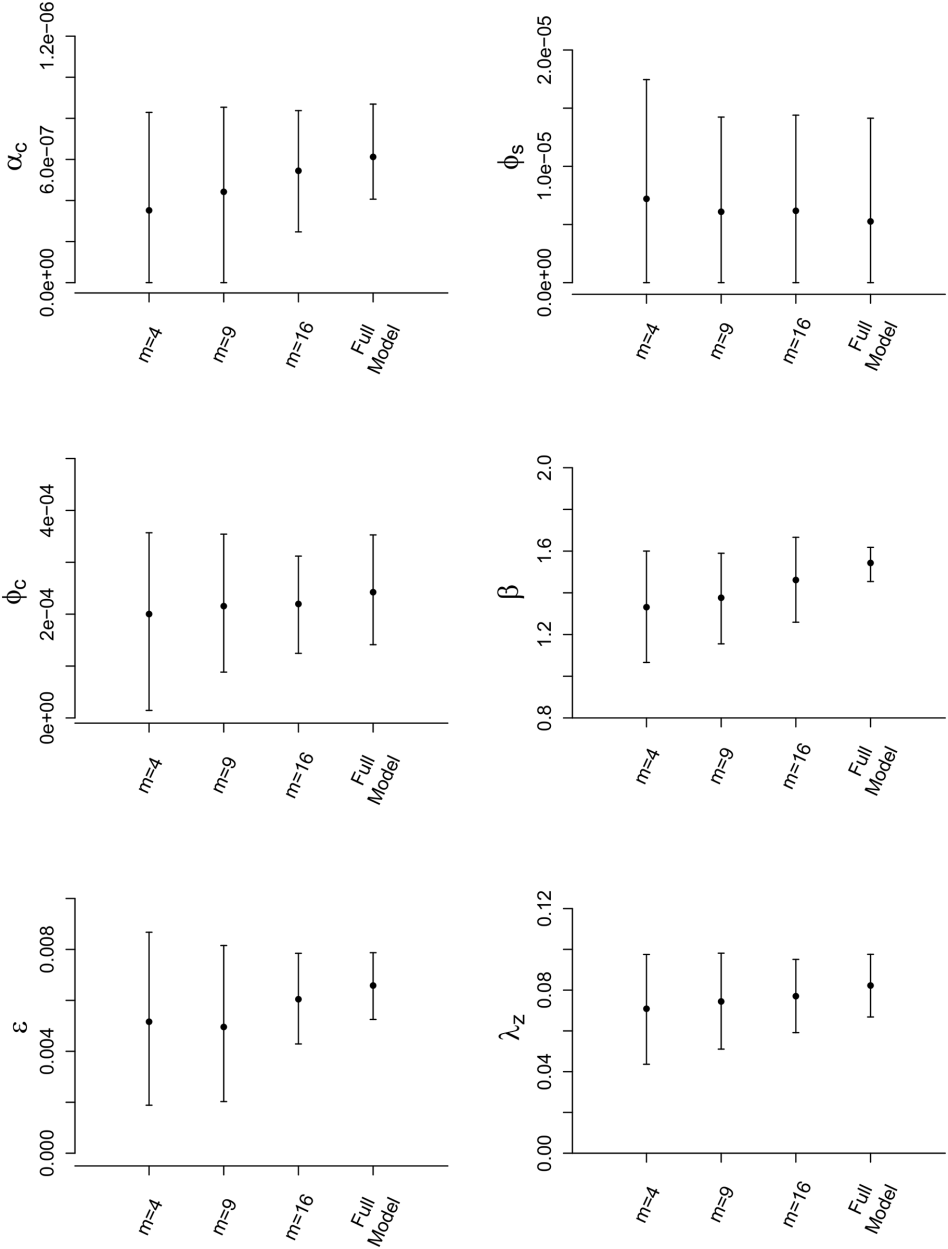
Posterior results for FMD-ILM using the spatial stratification method. Posterior means and 95% credible intervals for all parameters of the data augmented FMD-ILM under the spatial stratification method. We sampled *ρ* = 0.50 from each stratum. The results are compared to the full model.

Figs 4 and 5 show the results of implementing our sampling methods when fitting the data augmented FMD-ILM (tabulated results are given in S2 Table). Under the SRS approach, we see that posterior means for each ILM parameter tend to approach the posterior mean estimate under the full model as *ρ* is increased, similar to the findings of the simulation study. In general, we also observe that posterior variance decreases as *ρ* increases, resulting in tighter credible intervals closer to those under the full full model (which are generally the most narrow). Once again, these results mimic those seen in the simulation study.

Considering the spatially stratified schemes, we draw similar conclusions to those seen in the simulation study. Posterior accuracy tends to increase, and posterior variances decrease, as the number of strata, *m*, increases. In fact, for *m* = 16 we obtain posterior estimates that are very close to those seen under the full model for parameters *ϕ*_*s*_, *ϕ*_*c*_, and *λ*_*z*_. Credible interval widths for *ϕ*_*s*_ and *β* change negligibly with regards to choice of *m*. Further, although posterior variance decreases as *m* increases, the credible intervals obtained under SRS with *m* = 4 (the lowest number of strata) contain those seen under the full MCMC analysis, suggesting good approximate inference.

Comparing the two sampling schemes, we see that spatial stratification tends to yield more accurate results. For example, if we compare SRS at *ρ* = 0.50 with stratification even at *m* = 4 (also sampled with *ρ* = 0.50), we find that all parameters under the spatial stratification scheme provide very similar, or more accurate, posterior means and tighter credible intervals. As *m* increases, as we have also seen, these SRS-based results improve even further.

Once again, however, a key aspect in addition to modeling accuracy is reduction in computation time. Table 2 provides the run times (in hours) for each FMD data analysis. With the full model taking approximately 249.12 hours to run for 20,000 MCMC iterations, we notice significant time savings at *ρ* = 0.25 and *ρ* = 0.50 using SRS and at *m* = 4 using spatial stratification. The time savings are much lower and of more questionable benefit for larger *ρ* and *m*. Further, and once again, time savings achieved using these sampling methods would be expected to be greater for larger data sets (see discussion).

**Table 2:** Computation times for the FMD-ILM.

*ρ*	m	Computation Time (hours)
—	—	249.12
0.25	—	60.24
0.50	—	84.72
0.75	—	164.88
0.90	—	227.76
0.50	4	108.24
0.50	9	185.04
0.50	16	232.80

Computation run times (in hours) of fitting the data augmented FMD-ILM to the FMD data using various sampling techniques. Models produced 20,000 realizations from the posterior.

## Discussion

In this paper, we introduced sampling algorithms to help speed up the likelihood calculation for ILMs in a Bayesian MCMC framework. Unlike other proposed methods (e.g., [8, 12]), ours incorporates data augmented MCMC to allow for uncertainty about infection times into our analysis. We test the usefulness of our methods by comparing ILM parameter estimation under the full Bayesian analysis via a simulation study and using data from the 2001 FMD epidemic in the U.K. Our results show that overall, we were able to obtain fairly accurate (though less precise) results using sampling-based likelihood approximations compared to the results obtained under the full likelihood analysis. In terms of computation run times, we found significant savings could be made by using data sampling. Because the problem of repeated likelihood calculations under the full model is increased drastically with the inclusion of data augmentation, this is a result of key importance. However, we found using larger values of *ρ* or *m* can drastically reduce the time saving benefit over the full MCMC analysis.

In our studies, only two sampling techniques were considered. Possible future work could involve investigating other sampling procedures that might provide stronger inferential conclusions. For example, our spatial stratification technique consisted of dividing the population into equally sized cells/strata and then sampling from each cell with equal sampling proportions. This would seem intuitively sensible when the population is spread across a grid, as was the case in our simulation study. This may be reasonable for some crop diseases or perhaps if points on the grid represent regions or cells (e.g., consider the modeling of fire spread by [23]), but such a population layout would be quite unrealistic in most situations. (It was, of course, adequate for the main aim of this paper, which was to illustrate the facilitation of faster likelihood calculations via data sampling).

In most populations, some natural clustering of individuals tends to take place (e.g., there tend to be high density clusters of farms in regions where infrastructural and/or environmental conditions are suitable for the type of farming in question). In such situations, spatial strata could, for example, be based upon some spatial clustering method applied to the population data. Alternatively, for a population in which some sort of contact network, or series of such networks, were being used as a prime risk factor in the model, clustering based on the network(s), using say partitioning around medoids (PAM) [24], could be considered as a way of defining strata from which to sample.

Here, we assumed that the sampling proportion was invariant to time and/or stratum. However, it might be useful to allow the sampling proportion to vary according to one or both. We might also possibly want to place more weight on sampling at times when the epidemic intensity is highest. Alternatively, in the case of spatially stratified sampling, we might want to avoid sampling from some strata with very low epidemic intensity. Such methods could possibly provide faster computation concurrent with more accurate model parameterization.

There are also, of course, many other options for carrying out approximate inference when computational efficiency is a driving factor. For example, [25] use a Gaussian process emulator method based on mapping key summary statistics from model simulations to the parameter space. In a similar vein, the aforementioned so-called approximate Bayesian computational methods used by, for example, [14] and [15], can be employed. These are also based on comparing salient summary statistics from observed and simulated data. A systematic comparison of all of these different approaches would be of obvious interest.

Our study used a SIR modeling framework. We could extend the analysis presented here to a SEIR framework to investigate disease exposure times. In our modeling, we accounted for incubation by treating it as a period when infected individuals have not been diagnosed yet but can pass on the disease to others. Introducing an exposed state would indicate an individual has contracted the disease but cannot pass it on to others until they reach the infectious state, regardless of confirmation of disease diagnosis. Additionally, we assumed knowledge of when individuals were removed from the population; however, this would not be the case for most diseases (e.g., human influenza). In a future study, we can also explore scenarios where removal times are unknown and instead estimated through data augmentation. The modeling framework used in this paper was also set in discrete time. The time saving sampling used here can also be applied in a (more natural, arguably) continuous time modeling framework.

We have demonstrated as a proof of concept that, for these relatively small datasets, our sampling-based likelihood approximations can result in a significant decrease in computation time. The time savings using these sampling algorithms would be even more beneficial in large-scale problems involving massive data sets compared to a full Bayesian analysis. A natural avenue of possible future work would be to apply these techniques to much larger data sets. Of course, these techniques would only really be worth using for large data sets in which a full Bayesian analysis was computationally prohibitive, in which case the priority would likely to be to get some sort of ‘rough and ready’ inference done as quickly as possible, rather than worry too much about the quality of posterior approximation. However, some degree of thought would have to be given to the choice of *ρ* and the stratification methods used in order to achieve parametrization of a reasonable quality in a feasible time frame. Further work on the use of some sort of adaptive scheme, based initially on a quick pilot study over sampling proportions and stratification schemes, might also therefore be of interest.

## Supporting Information

S1 TableSummary statistics for the simulation studies.Summary statistics from the simulation studies comparing model parameter estimation across our different sampling schemes. The results are averaged over 10 different epidemics simulated from the data augmented spatial ILM with parameter values $\alpha =1.4,\beta =2.3,\lambda_{z}=\frac{1}{3}$, and *n* = 625. Here, CIs are the mean credible interval limits.(PDF)

S2 Table Summary statistics for modeling the FMD-ILM. Summary of results from fitting the data augmented FMD-ILM to the FMD data. We compare the results across our different sampling methods. Note that for spatial stratification, we sample *ρ* = 0.50 from each stratum.
(PDF)
