# Supplementary material for: Parameterizing Spatial Models of Infectious Disease Transmission that Incorporate Infection Time Uncertainty Using Sampling-Based Likelihood Approximations
Source: PLoS One. 2016 Jan 5;11(1):e0146253. doi: 10.1371/journal.pone.0146253 (PMC4701410; doi:10.1371/journal.pone.0146253)
Supplement: S1 Table — Summary statistics from the simulation studies comparing model parameter estimation across our different sampling schemes. The results are averaged over 10 different epidemics simulated from the data augmented spatial ILM with parameter values α=1.4,β=2.3,λz=13, and n = 625. Here, CIs are the mean credible interval limits. (PDF) [file pone.0146253.s001.pdf]

Parameterizing Spatial Models of Infectious Disease Transmission that Incorporate Infection Time Uncertainty Using Sampling-Based Likelihood Approximations

Rajat Malik[1], Rob Deardon[1,2,3], Grace P.S. Kwong[3]

1 Department of Mathematics & Statistics, University of Guelph, Guelph, Ontario, Canada
2 Faculty of Veterinary Medicine, University of Calgary, Calgary, Alberta, Canada
3 Department of Mathematics & Statistics, University of Calgary, Calgary, Alberta, Canada

Supporting Information

S1 Table. Summary statistics for the simulation studies.

Parameter	$\boldsymbol{\rho}$	m	$\mathbf{E}(\cdot\vert\mathbf{x})$	$\mathbf{V}(\cdot\vert\mathbf{x})$	Bias$[\mathbf{E}(\cdot\vert\mathbf{x})]$	95% CI
α	–	–	1.46	0.05	0.06	[1.05, 1.87]
	0.25	–	0.84	0.16	−0.61	[0.18, 1.64]
	0.50	–	1.09	0.10	−0.36	[0.51, 1.73]
	0.75	–	1.32	0.08	−0.14	[0.78, 1.89]
	0.90	–	1.45	0.08	0.05	[0.94, 2.01]
	0.10	4	1.24	0.18	−0.22	[0.49, 2.15]
	0.50	4	1.29	0.17	−0.17	[0.54, 2.12]
	0.10	9	1.24	0.09	−0.21	[0.75, 1.97]
	0.50	9	1.34	0.08	−0.12	[0.80, 1.89]
	0.10	16	1.39	0.06	−0.07	[0.96, 1.92]
	0.50	16	1.44	0.05	−0.02	[1.02, 1.88]
β	–	–	2.32	0.01	0.02	[2.13, 2.51]
	0.25	–	1.56	0.62	−0.75	[0.28, 3.10]
	0.50	–	1.64	0.30	−0.28	[0.67, 2.71]
	0.75	–	1.83	0.22	−0.49	[0.98, 2.76]
	0.90	–	2.24	0.21	−0.08	[1.35, 3.21]
	0.10	4	1.93	0.17	−0.39	[1.49, 2.48]
	0.50	4	2.07	0.06	−0.25	[1.62, 2.54]
	0.10	9	2.11	0.04	−0.21	[1.80, 2.52]
	0.50	9	2.26	0.03	−0.05	[1.95, 2.59]
	0.10	16	2.20	0.04	−0.12	[1.97, 2.64]
	0.50	16	2.37	0.02	0.04	[2.13, 2.62]
λ_z	–	–	0.33	1.09×10^{-3}	0.00	[0.27, 0.39]
	0.25	–	0.19	5.24×10^{-3}	−0.14	[0.06, 0.33]
	0.50	–	0.26	3.56×10^{-3}	−0.07	[0.15, 0.37]
	0.75	–	0.29	2.67×10^{-3}	−0.04	[0.19, 0.39]
	0.90	–	0.33	2.52×10^{-3}	1.43×10^{-3}	[0.23, 0.43]
	0.10	4	0.23	2.57×10^{-3}	−0.10	[0.15, 0.42]
	0.50	4	0.25	2.16×10^{-3}	−0.08	[0.17, 0.35]
	0.10	9	0.24	9.34×10^{-3}	−0.09	[0.18, 0.38]
	0.50	9	0.29	1.10×10^{-3}	−0.04	[0.23, 0.35]
	0.10	16	0.27	6.89×10^{-4}	−0.06	[0.22, 0.32]
	0.50	16	0.32	6.86×10^{-4}	-4.09×10^{-4}	[0.27, 0.37]
