# Supplementary material for: Parameterizing Spatial Models of Infectious Disease Transmission that Incorporate Infection Time Uncertainty Using Sampling-Based Likelihood Approximations
Source: PLoS One. 2016 Jan 5;11(1):e0146253. doi: 10.1371/journal.pone.0146253 (PMC4701410; doi:10.1371/journal.pone.0146253)
Supplement: S2 Table — Summary of results from fitting the data augmented FMD-ILM to the FMD data. We compare the results across our different sampling methods. Note that for spatial stratification, we sample ρ = 0.50 from each stratum. (PDF) [file pone.0146253.s002.pdf]

Parameterizing Spatial Models of Infectious Disease Transmission that Incorporate Infection Time Uncertainty Using Sampling-Based Likelihood Approximations

Rajat Malik[1], Rob Deardon[1,2,3], Grace P.S. Kwong[3]

1 Department of Mathematics & Statistics, University of Guelph, Guelph, Ontario, Canada
2 Faculty of Veterinary Medicine, University of Calgary, Calgary, Alberta, Canada
3 Department of Mathematics & Statistics, University of Calgary, Calgary, Alberta, Canada

Supporting Information

S2 Table. Summary statistics for modeling the FMD-ILM.

Parameter	$\boldsymbol{\rho}$	m	$\mathbf{E}(\cdot\|\mathbf{x})$	$\mathbf{V}(\cdot\|\mathbf{x})$	95% Credible Interval
α_c	–	–	6.12×10^{-7}	1.54×10^{-14}	$[4.06 \times 10^{-7}, 8.69 \times 10^{-7}]$
	0.25	–	4.87×10^{-7}	1.35×10^{-13}	$[2.75 \times 10^{-11}, 1.19 \times 10^{-6}]$
	0.50	–	4.27×10^{-7}	1.03×10^{-13}	$[1.20 \times 10^{-12}, 1.05 \times 10^{-6}]$
	0.75	–	4.19×10^{-7}	9.95×10^{-14}	$[9.30 \times 10^{-12}, 1.03 \times 10^{-6}]$
	0.90	–	4.46×10^{-7}	9.11×10^{-14}	$[2.99 \times 10^{-12}, 10 \times 10^{-6}]$
	0.50	4	3.52×10^{-7}	6.28×10^{-14}	$[1.17 \times 10^{-10}, 8.29 \times 10^{-7}]$
	0.50	9	4.42×10^{-7}	5.84×10^{-14}	$[4.95 \times 10^{-11}, 8.54 \times 10^{-7}]$
	0.50	16	5.45×10^{-7}	2.27×10^{-14}	$[2.47 \times 10^{-7}, 8.37 \times 10^{-7}]$
ϕ_s	–	–	5.26×10^{-6}	1.98×10^{-11}	$[6.21 \times 10^{-11}, 1.41 \times 10^{-5}]$
	0.25	–	1.34×10^{-5}	1.04×10^{-10}	$[1.12 \times 10^{-9}, 3.30 \times 10^{-5}]$
	0.50	–	1.12×10^{-5}	7.22×10^{-11}	$[1.56 \times 10^{-9}, 2.78 \times 10^{-5}]$
	0.75	–	7.55×10^{-6}	3.27×10^{-11}	$[8.97 \times 10^{-11}, 1.86 \times 10^{-5}]$
	0.90	–	8.64×10^{-6}	4.15×10^{-11}	$[3.91 \times 10^{-10}, 2.10 \times 10^{-5}]$
	0.50	4	7.21×10^{-6}	2.84×10^{-11}	$[7.21 \times 10^{-11}, 1.75 \times 10^{-5}]$
	0.50	9	6.10×10^{-6}	1.89×10^{-11}	$[2.04 \times 10^{-9}, 1.42 \times 10^{-5}]$
	0.50	16	6.18×10^{-6}	1.90×10^{-11}	$[7.48 \times 10^{-10}, 1.44 \times 10^{-5}]$
ϕ_c	–	–	2.42×10^{-4}	3.03×10^{-9}	$[1.41 \times 10^{-4}, 3.53 \times 10^{-4}]$
	0.25	–	1.70×10^{-4}	1.63×10^{-8}	$[4.35 \times 10^{-9}, 4.17 \times 10^{-4}]$
	0.50	–	1.99×10^{-4}	2.04×10^{-8}	$[7.57 \times 10^{-9}, 4.69 \times 10^{-4}]$
	0.75	–	1.86×10^{-4}	1.13×10^{-8}	$[4.12 \times 10^{-8}, 3.71 \times 10^{-4}]$
	0.90	–	2.12×10^{-4}	1.29×10^{-8}	$[2.17 \times 10^{-7}, 4.06 \times 10^{-4}]$
	0.50	4	20×10^{-4}	7.83×10^{-9}	$[1.45 \times 10^{-5}, 3.57 \times 10^{-4}]$
	0.50	9	2.16×10^{-4}	4.66×10^{-9}	$[8.83 \times 10^{-5}, 3.54 \times 10^{-4}]$
	0.50	16	2.20×10^{-4}	2.32×10^{-9}	$[1.24 \times 10^{-4}, 3.12 \times 10^{-4}]$
β	–	–	1.54×10^{-0}	1.79×10^{-3}	$[1.45 \times 10^{-0}, 1.62 \times 10^{-0}]$
	0.25	–	1.11×10^{-0}	6.02×10^{-2}	$[6.33 \times 10^{-1}, 1.59 \times 10^{-0}]$
	0.50	–	1.18×10^{-0}	3.22×10^{-2}	$[8.29 \times 10^{-1}, 1.53 \times 10^{-0}]$
	0.75	–	1.34×10^{-0}	2.71×10^{-2}	$[1.01 \times 10^{-0}, 1.66 \times 10^{-0}]$
	0.90	–	1.41×10^{-0}	7.95×10^{-3}	$[1.24 \times 10^{-0}, 1.59 \times 10^{-0}]$
	0.50	4	1.33×10^{-0}	1.85×10^{-2}	$[1.07 \times 10^{-0}, 1.60 \times 10^{-0}]$
	0.50	9	1.38×10^{-0}	1.23×10^{-2}	$[1.16 \times 10^{-0}, 1.59 \times 10^{-0}]$
	0.50	16	1.46×10^{-0}	1.10×10^{-2}	$[1.26 \times 10^{-0}, 1.67 \times 10^{-0}]$
ϵ	–	–	6.58×10^{-3}	4.46×10^{-7}	$[5.25 \times 10^{-3}, 7.87 \times 10^{-3}]$
	0.25	–	2.41×10^{-3}	3.29×10^{-6}	$[5.37 \times 10^{-7}, 5.92 \times 10^{-3}]$
	0.50	–	2.90×10^{-3}	4.04×10^{-6}	$[1.19 \times 10^{-7}, 6.63 \times 10^{-3}]$
	0.75	–	3.40×10^{-3}	3.41×10^{-6}	$[1.51 \times 10^{-6}, 6.61 \times 10^{-3}]$
	0.90	–	5.57×10^{-3}	9.97×10^{-7}	$[3.62 \times 10^{-3}, 7.52 \times 10^{-3}]$
	0.50	4	5.16×10^{-3}	2.99×10^{-6}	$[1.88 \times 10^{-3}, 8.67 \times 10^{-3}]$
	0.50	9	4.96×10^{-3}	2.41×10^{-6}	$[2.03 \times 10^{-3}, 8.15 \times 10^{-3}]$
	0.50	16	6.04×10^{-3}	8.20×10^{-7}	$[4.29 \times 10^{-3}, 7.85 \times 10^{-3}]$
λ_z	–	–	8.23×10^{-2}	6.21×10^{-5}	$[6.68 \times 10^{-2}, 9.76 \times 10^{-2}]$
	0.25	–	2.93×10^{-2}	4.74×10^{-4}	$[1.73 \times 10^{-7}, 7.11 \times 10^{-2}]$
	0.50	–	5.43×10^{-2}	6.10×10^{-4}	$[4.70 \times 10^{-3}, 9.97 \times 10^{-2}]$
	0.75	–	5.02×10^{-2}	2.56×10^{-4}	$[1.90 \times 10^{-2}, 8.22 \times 10^{-2}]$
	0.90	–	7.37×10^{-2}	4.26×10^{-4}	$[3.19 \times 10^{-2}, 1.12 \times 10^{-1}]$
	0.50	4	7.09×10^{-2}	1.92×10^{-4}	$[4.36 \times 10^{-2}, 9.75 \times 10^{-2}]$
	0.50	9	7.44×10^{-2}	1.44×10^{-4}	$[5.11 \times 10^{-2}, 9.81 \times 10^{-2}]$
	0.50	16	7.70×10^{-2}	8.39×10^{-5}	$[5.91 \times 10^{-2}, 9.51 \times 10^{-2}]$
